# Supplementary material for: Non-adherence to long-lasting insecticide treated bednet use following successful malaria control in Tororo, Uganda
Source: PLoS One. 2020 Dec 3;15(12):e0243303. doi: 10.1371/journal.pone.0243303 (PMC7714220; doi:10.1371/journal.pone.0243303)
Supplement: S1 File — (DOC) [file pone.0243303.s001.doc]

| **MOSQUITO COLLECTION AND LLIN ADHERENCE FORM (attached additional form if needed)** | | | Number of rooms used for sleeping tonight |___|___| | | | | | | | | |
| --- | --- | --- | --- | --- | --- | --- | --- | --- | --- | --- | --- |
|  |  |  | Number of rooms used for sleeping tonight that have windows \|___\|___\| | | | | | | | | |
| Time LT set |___|___| : |___|___| (24 hr scale) | | | | | Time LT collected |___|___| : |___|___| (24 hr scale) | | | | | | |
| Room number (list all rooms where traps were set) |  |  | |  | |  |  |  |  |  |  |
| Whether LT was working the following morning | □ Yes □ No | □ Yes □ No | | □ Yes □ No | | □ Yes □ No | □ Yes □ No | □ Yes □ No | □ Yes □ No | □ Yes □ No | □ Yes □ No |

| Cohort participant ID | 3 _ _ _ | 3 _ _ _ | 3 _ _ _ | 3 _ _ _ | 3 _ _ _ | 3 _ _ _ | 3 _ _ _ | 3 _ _ _ |
| --- | --- | --- | --- | --- | --- | --- | --- | --- |
| Did they sleep in the house last night? If no, skip remaining questions | □ Yes □ No | □ Yes □ No | □ Yes □ No | □ Yes □ No | □ Yes □ No | □ Yes □ No | □ Yes □ No | □ Yes □ No |
| Room number / Sleeping area (letter) | **/** | / | / | / | / | / | / | / |
| Did they or someone else report them sleeping under an LLIN last night? If no, skip next question | □ Yes □ No  □ Unknown | □ Yes □ No  □ Unknown | □ Yes □ No  □ Unknown | □ Yes □ No  □ Unknown | □ Yes □ No  □ Unknown | □ Yes □ No  □ Unknown | □ Yes □ No  □ Unknown | □ Yes □ No  □ Unknown |
| Was an LLIN observed hanging above sleeping area? | □ Yes □ No | □ Yes □ No | □ Yes □ No | □ Yes □ No | □ Yes □ No | □ Yes □ No | □ Yes □ No | □ Yes □ No |
| Approximate time went to bed (skip if unknown) | **:** | **:** | **:** | **:** | **:** | **:** | **:** | **:** |
| Approximate time got out of bed (skip if unknown) | **:** | **:** | **:** | **:** | **:** | **:** | **:** | **:** |

Complete section below after assessing mosquitoes collected from household

| Room number |  |  |  |  |  |  |  |  |  |
| --- | --- | --- | --- | --- | --- | --- | --- | --- | --- |
| Type of collection (circle one per column)* | LT / RC / ET | LT / RC / ET | LT / RC / ET | LT / RC / ET | LT / RC / ET | LT / RC / ET | LT / RC / ET | LT / RC / ET | LT / RC / ET |
| Total blood fed *An. gambiae sl* |  |  |  |  |  |  |  |  |  |
| Total unfed *An. gambiae sl* |  |  |  |  |  |  |  |  |  |
| Total gravid/semigravid *An. gambiae sl* |  |  |  |  |  |  |  |  |  |
| Total *An. gambiae sl* unable to assess |  |  |  |  |  |  |  |  |  |
| Total blood fed *An. funestus* |  |  |  |  |  |  |  |  |  |
| Total unfed *An. funestus* |  |  |  |  |  |  |  |  |  |
| Total gravid/semigravid *An. funestus* |  |  |  |  |  |  |  |  |  |
| Total *An. funestus* unable to assess |  |  |  |  |  |  |  |  |  |
| Total blood fed other Anopheles sp. |  |  |  |  |  |  |  |  |  |
| Total unfed other Anopheles sp. |  |  |  |  |  |  |  |  |  |
| Total gravid/semigravid other Anopheles sp. |  |  |  |  |  |  |  |  |  |
| Total other Anopheles sp. unable to assess |  |  |  |  |  |  |  |  |  |
| Total number dissected for parity |  |  |  |  |  |  |  |  |  |
| Total number parous |  |  |  |  |  |  |  |  |  |
| Total number nulliparous |  |  |  |  |  |  |  |  |  |
| Total Culex |  |  |  |  |  |  |  |  |  |

* LT = CDC light trap; RC = Resting collections; ET = Exit traps
