# Supplementary material for: Non-adherence to long-lasting insecticide treated bednet use following successful malaria control in Tororo, Uganda
Source: PLoS One. 2020 Dec 3;15(12):e0243303. doi: 10.1371/journal.pone.0243303 (PMC7714220; doi:10.1371/journal.pone.0243303)
Supplement: S2 File — Exit interview questionnaire relating to bednet adherence. (DOCX) [file pone.0243303.s002.docx]

# S1: EXIT INTERVIEW QUESTIONNAIRE RELATING TO BEDNET ADHERENCE

**PRISM/Cohort Study: Verbal Consent for Exit Interview About Bednet Use**

Script in *italics*

*Hello, my name is ____________________________ (print name of interviewer). I’m working with the Infectious Diseases Research Collaboration as a study clinician. We are conducting exit interviews for all participants who have been in the cohort study for the last 2 years. We are interested in understanding your thoughts about bed net use in your home and community. and would very much appreciate your participation in this survey. This information will help the government to plan malaria control services and for us to plan future studies related to bed net use in the country.*

*As part of the interview, we would like to ask some questions about your understanding of bed net use, its importance and any challenges you face with bed net use.*

*These questions will take about 30 minutes to complete. Whatever information you provide will be kept confidential, and will not be shared with anyone other than members of the study team. Participation in this interview is voluntary and if we should come to any question you don’t want to answer, just let me know and I will go onto the next question; or you can stop the interview at any time. However, we hope you will participate in the interview since your views are important.*

*At this time, do you want to ask me anything about the survey? May I begin the interview now?*

Participant decision: Refused interview Accepts interview

Signature of interviewer: ____________________________________ Date: ________________

| **PRISM Exit Interview Bednet Adherence** | | | |
| --- | --- | --- | --- |
| **Variable Name** | **Question** | **Variable Type** | **Variable Codes** |
| **Section 1: Identification** |  |  |  |
| intnum | Interviewer's Number | Numeric |  |
| date | Date of interview | DateTime | dd/mm/yy |
| hhid | Household ID | Numeric |  |
| cohortid | Cohort ID | Numeric | 3001-3900 |
| **Why net might not be used** | | | |
| Which of these are important reasons that people do not hang/mount their bednets if they own them? | | | |
| nospace | ...Nowhere to hang | Numeric | 1 - Yes 0 - No |
| howhang | ...Don't know how to hang net | Numeric | 1 - Yes 0 - No |
| notools | ...No tools to hang net | Numeric | 1 - Yes 0 - No |
| nofit | ...Shape or size did not fit | Numeric | 1 - Yes 0 - No |
| dontlike | ...Don't like bednets | Numeric | 1 - Yes 0 - No |
| noneed | …Don't need a bednet | Numeric | 1 - Yes 0 - No |
| extranet | ...Extra net/Used for Visitor/ Owner not home | Numeric | 1 - Yes 0 - No |
| oldnet | …net is old/dirty/has holes/infested with bugs | Numeric | 1 - Yes 0 - No |
| otherhung | Specify other reason why a net might not be hung up/mounted | String |  |
|  |  |  |  |
|  | Which of these are important reasons why someone might not use a bednet that is already hung above a sleeping space? | | |
| nutoohot | ...Too hot | Numeric | 1 - Yes 0 - No |
| nusmell | ...Don't like smell | Numeric | 1 - Yes 0 - No |
| nunomos | ...No mosquitoes | Numeric | 1 - Yes 0 - No |
| nuold | ...Net too old/too many holes | Numeric | 1 - Yes 0 - No |
| nunothung | ...Net not hung | Numeric | 1 - Yes 0 - No |
| nudirty | ...Net too dirty | Numeric | 1 - Yes0 - No |
| nunotkillins | ...Net no longer kill insects | Numeric | 1 - Yes 0 - No |
| nunomal | …no malaria | Numeric | 1 - Yes 0 - No |
| nuforgot | …Forgot | Numeric | 1 - Yes 0 - No |
| nuknowhow | …don't know how to use | Numeric | 1 - Yes 0 - No |
| nuother | ...Other | Numeric | 1 - Yes 0 - No |
| othrnt | Specify other reason why a net might not be used | String |  |
| **Understanding of malaria and malaria risk** | | | |
| stillmalaria | Do you think that malaria is a risk to you or your family? | Numeric | 1 - Yes 0 - No |
| changerisk | Do you think that the risk of malaria in this community has changed over the last year? | Numeric | 1 - Yes 0 - No |
| howchange | If the risk of malaria has changed, do you think the risk of malaria has gotten worse or better? | Numeric | 1 - Less risk 0 - More risk -7: Don't know |
|  | If you think the risk of malaria has changed, which of these are important ways that you learned that information? | | |
| learnprovider | …my health provider told me | Numeric | 1 - Yes 0 - No |
| learnfamily | …close family told me | Numeric | 1 - Yes 0 - No |
| learnleader | …village leaders told me | Numeric | 1 - Yes 0 - No |
| learnradio | …heard it on radio or in newspapers | Numeric | 1 - Yes 0 - No |
| learnmyself | …figured it out myself | Numeric | 1 - Yes 0 - No |
| learnsick | …no one gets sick with malaria anymore | Numeric | 1 - Yes 0 - No |
| learnfriends | …a friend or friends told me | Numeric | 1 - Yes0 - No |
| learnother | Please specify other ways you may have heard that the risk of malaria has changed | String |  |
|  | Which of these are ways that malaria is transmitted? | | |
| walkrain | …walking in rain | Numeric | 1 - Yes 0 - No |
| coughing | …caught from other's coughing | Numeric | 1 - Yes 0 - No |
| mosquito | …from the bite of a mosquito | Numeric | 1 - Yes 0 - No |
| housefly | …From the bite of housefly | Numeric | 1 - Yes 0 - No |
| badfood | …from eating spoiled/bad food | Numeric | 1 - Yes 0 - No |
| unripefruits | …eating unripe fruits | Numeric | 1 - Yes 0 - No |
| othrnt | Specify other ways malaria is transmitted | String |  |
|  | Which of these are ways that bednets protect against malaria? | | |
| killmosquitoes | …bednets kill mosquitoes | Numeric | 1 - Yes 0 - No |
| repelmosquitoes | …mosquitoes stay away from houses that use bednets | Numeric | 1 - Yes 0 - No |
| blockbites | …bednets block mosquitoes from biting sleeping people | Numeric | 1 - Yes 0 - No |
| otherprotect | Specify other ways that bednets protect against malaria | String |  |
|  | Among which of these groups is it important that they use bednets every night? | | |
| useadults | …Adults | Numeric | 1 - Yes 0 - No |
| usechildren | …children under five years | Numeric | 1 - Yes 0 - No |
| useschool | …school age children | Numeric | 1 - Yes 0 - No |
| useolder | …older/elderly adults | Numeric | 1 - Yes 0 - No |
| usepregnant | …pregnant women | Numeric | 1 - Yes 0 - No |
| useall | …everyone in the household | Numeric | 1 - Yes0 - No |
| useother | specify other important groups that should use bednets | String |  |
| **Benefits and risks of bednet use** | | | |
|  | Which of these are major benefits of bednet use? | | |
| keepswarm | ….keeps people warm at night | Numeric | 1 - Yes 0 - No |
| blocksmosquitoes | …blocks annoying buzzing and bites from mosquitoes and other insects | Numeric | 1 - Yes 0 - No |
| protectsmalaria | …protects against malaria | Numeric | 1 - Yes 0 - No |
| notuseful | …I don't think bednets are useful | Numeric | 1 - Yes 0 - No |
| otherbenefit | please specify other benefits of bednet use | String |  |
| knowhow | Do you feel that you know how to use a net correctly? | Numeric | 1 - Yes 0 - No -9: Skipped |
| costnonuse | Rather than helping you, do you think there are any ways that using a net can cause you or others harm or sickness? | Numeric | 1 - Yes 0 - No -9: Skipped |
|  | If you think using a bednet can cause harm/illness, which of these groups should avoid or be careful using a bednet? | | |
| avoidchildren | ….children | Numeric | 1 - Yes 0 - No |
| avoidadults | …adults | Numeric | 1 - Yes 0 - No |
| avoidpregnant | …pregnant women | Numeric | 1 - Yes 0 - No |
| avoidother | specify other groups who should avoid/or be careful when using a bednet | Numeric | 1 - Yes 0 - No |
| netharm | If you think a bednet can cause harm, please specify some harms using a net can cause: | String |  |
| **Motivations for bednet use** | | | |
|  | If you use a bednet or have used a bednet in the past, which of these are some of the important reasons you used it? | | |
| pastmalaria | …i have had malaria before and want to prevent it | Numeric | 1 - Yes 0 - No |
| fearmalaria | …I have seen people get ill from malaria and want to prevent it | Numeric | 1 - Yes 0 - No |
| routineuse | …I use it regularly as a habit, no particular reason | Numeric | 1 - Yes 0 - No |
| everyoneuses | …I use bednets because everyone I know uses them | Numeric | 1 - Yes 0 - No |
| helpcommunity | …using bednets protects me and helps the community | Numeric | 1 - Yes 0 - No |
| provideradvice | …because a doctor/nurse/health provider told me to use a bednet | Numeric | 1 - Yes 0 - No |
| othermotivate | specify other important reasons that lead you to use bednets | String |  |
| safeuse | do you think bednets are safe to use? | Numeric | 1 - Yes 0 - No |
| famnorm | Do you think most of your close family/friends use bednets? | Numeric | 1 - Yes 0 - No -9: Skipped |
| commnorm | Do you think most people in your community use bednets? | Numeric | 1 - Yes 0 - No -9: Skipped |
| **Challenges of bednet use** | | | |
|  | Which of these are important challenges that might prevent you or someone in your household from using a bednet every night? | | |
| forgetuse | …easy to forget to use | Numeric | 1 - Yes 0 - No |
| getsinway | …bednets get in the way, inconvenient | Numeric | 1 - Yes 0 - No |
| toohot | …bednets are too hot | Numeric | 1 - Yes 0 - No |
| infested | …bednets get infested with bedbugs or other insects | Numeric | 1 - Yes 0 - No |
| dirtynet | …bednets get dirty and people don't want to use dirty nets | Numeric | 1 - Yes 0 - No |
| holesnet | …bednets get holes and people don't want to use nets with holes | Numeric | 1 - Yes 0 - No |
| hardmount | …bednets are difficult to mount/hang | Numeric | 1 - Yes 0 - No |
| otherchallenge | Please specify other challenges to using bednets for you or your household | String |  |
| risknouse | What is the risk of sleeping a night without any bednet protection? | Numeric | Not using a bednet is... 1 - very risky 2 - somewhat risky 3 - doesn't make a difference 4 - just a little risky 5 - not at all risky -7: Don't know |
| **Coping and behavioral skills of bednet use** | | | |
| easyuse | How easy are bednets to use? | Numeric | Bednets are ... 1 - very easy to use 2 - somewhat easy to use 3 - neither easy nor difficult 4 - just a little difficult to use 5 - very difficult to use -7: Don't know |
| easyroutine | How easy is it for you to make a habit out of using a bednet? | Numeric | 1 - very easy  2 - somewhat easy  3 - neither easy nor difficult 4 - just a little difficult  5 - very difficult  -7: Don't know |
| copeholes | Does a bednet still provide protection if there are holes in it? | Numeric | 1 - Yes 0 - No -9: Skipped |
| useholes | Do you think most people keep using a bednet if it has holes in it? | Numeric | 1 - Yes 0 - No -9: Skipped |
| youdoholes | What do you or your household do if there are holes in your bednet? | Numeric | 1 - Sew them myself 2 - Bring to a tailor to sew 3 - Use anyways 4 - Store it away 5 - Replace the net with another one -7: Never happened to me |
| copedirty | Does a bednet still provide protection if it is soiled/dirty? | Numeric | 1 - Yes 0 - No -9: Skipped |
| usedirty | Do you think most people keep using a bednet if it is dirty? | Numeric | 1 - Yes 0 - No -9: Skipped |
| youdodirty | What do you or your household do if your bednet is dirty? | Numeric | 1 - Wash it myself 2 - Have someone else wash it 3 - Use anyways 4 - store it away 5 - Replace the net with another one -7: Never happened to me |
| copebugs | Does a bednet still provide protection if it is infested with bedbugs/insects? | Numeric | 1 - Yes 0 - No -9: Skipped |
| usebugs | Do you think most people keep using a bednet if it is infested with bedbugs/insects? | Numeric | 1 - Yes 0 - No -9: Skipped |
| youdobugs | What do you or your household do if there are bedbugs/insects infesting your net? | Numeric | 1 - Put it out in the sun 2 - Wash it well 3 - Use smoke 4 - Not use the net 5 - Replace the net -7: Don't know/never happened to me |
| effectivebednet | If you used a bednet over the last month, how effective (how well is it working?) do you think your bednet is? | Numeric | My bednet is...  1 - very effective 2 - somewhat effective 4 - just a little effective 5 - not at all effective -7: Don't know |
| **Perceptions of community norms** | | | |
| selfappraisal | Do you think your bednet use over the last month was good enough to protect you from malaria? | Numeric | 1 - Yes 0 - No -9: Skipped |
| householdassess | Do you think your bednet use was similar to other members of your household? | Numeric | 1 - Yes 0 - No -9: Skipped |
| commassess | Do you think your bednet use was similar to most people in your community? | Numeric | 1 - Yes 0 - No -9: Skipped |
| commrank | Do you think your bednet use over the last month was better, same or worse than most people in your community? | Numeric | 2 -Better 1 - Same 0 - Worse -9: Skipped |
| **Last use episode** | |  |  |
| nonuseepisode | Please bring to mind the last night you recall not using a bednet. Now, can you tell me why you did not use a bednet during that night? | Numeric | 1 - I just forgot 2 - too hot 3 - No mosquitoes around 4 - No malaria around 5 - Never miss a night 5 - Was travelling/did not sleep at home -7: Not sure |
| **Interviewer Details** | |  |  |
| comments | Interviewer's Comments | String |  |
| stoptime | End time of interview | DateTime |  |
