# Supplementary material for: Non-adherence to long-lasting insecticide treated bednet use following successful malaria control in Tororo, Uganda
Source: PLoS One. 2020 Dec 3;15(12):e0243303. doi: 10.1371/journal.pone.0243303 (PMC7714220; doi:10.1371/journal.pone.0243303)
Supplement: Attachment — Submitted filename: Response to reviewers.docx [file pone.0243303.s004.docx]

“**Non-adherence to long-lasting insecticide treated bednet use following successful malaria control in Tororo, Uganda**”.

Response to Reviewers:

**Reviewer #1:**

1) Line 221: It should be Result section, please change

*As requested, we have corrected this typo and removed “and discussion” from line 221.*

2) Line 235: Authors could discuss more rigorously regarding the findings "general compliance with LLIN decreases during periods of lower mosquito density and increases during periods of higher mosquito density" in line with the findings (line 303): the reason one might not use LLIN is 28.8 % answered that there were no mosquitoes compared to 'too hot' (85.6%). Please explain… considering understanding the importance of LLIN compliance is strongly associated with a higher likelihood of malaria

*Thank you for highlighting this important behavioral point that highlights individuals’ reported reasons for the observed behaviors. We have edited line 235 to read:*

*“In this study, it appears that perceptions of risk of malaria transmission decreased as community burden and mosquito densities declined. Along with this decrease in actual and perceptions of risk, it is possible that other salient barriers to use, such as the heat (referenced by 85.6% of respondents), bedbugs (24.6%), not liking the smell (11.5%) or the bednet being too dirty (9.6%), overcome other incentives to consistently use LLINs and lead to lower adherence.”*

3) Line 318: Author should add in the discussion section regarding non-adherence the used of LLINs in the age group under 5 years old. Considering the age range is still under the supervision of parents or adults, therefore I was clarified on my previous review note why was age range under 5 years old, included.

This is another important point and we agree that more discussion of the intrahousehold behaviors related to age and bednet use is warranted. We have added the following text on line 318:

“*This is particular interesting given that it might be expected that children under five will often use their bednets under the supervision of adults in the household. The discordance between LLIN use by adults and young children deserves special attention in future efforts to understand who is responsible for choices related to bednet use in Ugandan households*.”
